# Supplementary material for: “If I am alive, I am happy”: Defining quality of care from the perspectives of key maternal and newborn health stakeholders in Papua New Guinea
Source: PLOS Glob Public Health. 2024 May 21;4(5):e0002548. doi: 10.1371/journal.pgph.0002548 (PMC11108204; doi:10.1371/journal.pgph.0002548)
Supplement: S1 Text — (DOCX) [file pgph.0002548.s002.docx]

**Gender Diversity**

The authors would like to acknowledge that this project, as well as most of the available evidence in this field drawn on, is derived from the study of cisgender women, and that cisgender women, transgender men, nonbinary, gender fluid and intersex individuals can become pregnant and should be involved in defining quality of pregnancy care. To facilitate readability and be concise, the term ‘woman’ is used to refer to all people who may become pregnant, however the terms ‘client’ and ‘patient’ are also used throughout. It is important to consider the perspectives of gender diverse individuals when discussing pregnancy care to promote inclusivity and prevent discrimination.
