# Supplementary material for: “If I am alive, I am happy”: Defining quality of care from the perspectives of key maternal and newborn health stakeholders in Papua New Guinea
Source: PLOS Glob Public Health. 2024 May 21;4(5):e0002548. doi: 10.1371/journal.pgph.0002548 (PMC11108204; doi:10.1371/journal.pgph.0002548)
Supplement: S2 Table — (DOCX) [file pgph.0002548.s004.docx]

**Interview Guide**

**Parent Interviews**

| **General Questions About** | |
| --- | --- |
| - Socio-demographics (gender, age, marital status, highest level of education completed) - General health (any co-morbidities – specifically ask about malaria, TB, nutrition deficiencies) - Reproductive history (number of pregnancies and location of birth including times did not give birth to a baby, how many times given birth to a baby that lived, was stillborn or died after birth, how many children given birth to who are now alive including most recent childbirth, number of previous induced or spontaneous abortions, number of previous caesarean sections) | |
| **In-depth Interview Question** | **Probe/Follow Up** |
| Can you please tell me about your  labour experience, from when it began  to when and where you delivered your  baby, including how you travelled to get  to this facility  *NB: Some questions below will be*  *skipped, or simply clarified if the woman*  *has already explained specific scenarios.* | - i.e., time, distance, mode, travel at night/day, who assisted/accompanied - Was the woman asked to bring someone with you to be present in the labour? (For adolescents ask if this was a requirement) - Is this the first time at this facility? i.e., familiarity with facility and providers - How did she know she was in labour? - Discuss decision to come to a facility, who made this decision and what influenced it - Was this always the plan? Or did you make this plan at the last minute? - Why did you come to this facility? |
| For this labour, did you come directly to  xxx (birth facility) when you gave birth to  BABY NAME or did you go to another  health facility first? | - If yes, why? i.e., referral, reason for referral? |
| If another facility visited first: can you  tell me what happened at that first  facility  Can you tell me about what happened  when you reached this facility? | - i.e., waiting time, availability of staff, medical examination, who conducted check-ups, diagnostic services, cost, behaviour of staff, cleanliness of surrounding, information sharing, privacy - For adolescent women: any procedures or examinations? Was the adolescent asked to consent for these? Did they ask someone else’s permission, e.g., guardian/parent? |
| Tell us about what happened when you  were taken to the labour room? | - i.e., Who was present? Who did what? Was a health worker present when the baby came out? - i.e., entry to labour room, promptness of care, pain management, access to food and fluids, communication and information sharing, cleanliness of labour room, privacy |
| Can you tell us a bit about your labour  and childbirth? | - Any support person present? How accepting was the facility of this support person? Whose choice was it to have this support person? - What were her expectations of the male partner if she has one? Was he there? Should he be there? Did he want to be? Any barrier faced for him to be there? - How did it compare to what was expected? - Elaborate on what was the same or different to expectations - Method? (Vaginal, Caesarean, Unknown) - Any interventions i.e., episiotomy, cutting, forceps, vacuum - Number of babies - What position for the birth? Was this your choice? Was there any suggestion made about a different position that might be possible? - Companion in labour (family member) |
| Are you currently breastfeeding? | - Yes/No/Unknown - Time between birth and babies’ first attempt at breastfeeding. - Who helped the mother with supporting the baby to breastfeed? - Is the mother and baby able to breastfeed now - Any difficulties already overcome, still ongoing? |
| Do you plan to breastfeed once you go  home? | - If not first child, ask about previous success / - challenges with feeding - Does she know of any problems women can face with breastfeeding even if she has not experienced them herself? - What would she do if she was finding it hard or painful? Is there someone she would seek advice from? |
| We are interested in how women experience care during childbirth. Can you tell me how you felt the midwives and other facility staff behaved towards you?  What did they do well?  Was there anything that you wished  they had done differently? | - Any examples of how the staff were helpful or made the woman feel comfortable? - Ask about the communication from staff – did she feel well informed? Able to ask questions? - Is there anything a staff member did or said that was useful and should happen for other women in labour? - Any ideas about if something could be different next time about her interactions with the staff, what would it be? - If she were advising a friend how to prepare for labour in this facility, what would she want that friend to know? - Treatment by staff – Depending on previous responses and the interactions between researcher and participant, some questions will be carefully raised to ask about any disrespectful or abusive behaviour during the labour - Were there any incidents of shouting or screaming? Were there any incidents of stigma and discrimination? |
| Did any of the health staff examine you  internally by putting their fingers in your  vagina? | - Ask about the value mother places on vaginal examination, e.g., to inform on progress or likelihood of complications - Ask about how was permission obtained, was private health information discussed in a way that others could hear, were vaginal examinations conducted in a way that others could see, did you feel exposed? |
| Can you tell us about what happened  after you gave birth? | - Monitoring by staff - Regular check-ups and observations - Complications and responsiveness of staff - Medicines and other supplies, food, cost - Behaviour of staff - Cleanliness - Availability of water, electricity - Counselling/clarifications provided - Privacy - Emotional support |
| What did you pay for your stay at the  facility? Was this expected / did you  know about this before coming to the  facility?  Were there any other costs or  requirements other than this money  amount you have mentioned? | - What is the standard advertised fee for this facility? - Was this amount she paid different to this? - Did staff suggest or ask for a bribe, informal payment, or gift? |
| What is the most important concern to you during childbirth? | - In your opinion, what could be the important needs of a labouring women? |
| What happened at the time of  discharge? | - Any payment made? Sign any forms? - Birth registration? - Vaccination? - Other further health care for mother or baby - provided? If so, by who and what was - explained? - Any take-home information given? (i.e. - pamphlets etc) Did you understand what was - communicated? |
| How was your overall experience at the facility? | - What did you appreciate most at the facility? What did you find most disappointing? Do you have any suggestions for improvement? |

**Healthcare professional, healthcare manager, and administrator interviews**

| **General Questions About** | |
| --- | --- |
| - Socio-demographics, qualifications, and experience (gender, age, highest level of education completed, qualifications) | |
| **In-depth Interview Question** | **Probe/Follow Up** |
| Tell me about your work experience in maternal and newborn health care in PNG? | - Years of experience in PNG health system - Years of experience in maternal and newborn care - Years at this facility |
| Tell me about your current role at the facility/in the health system? | - Role and responsibilities - How many years worked at facility/in the health system (i.e., for health planners) - Different areas of work and frequency (labour ward, postnatal, other) - What are common complications at your facility? - What can be managed here and what might need to be referred for specialist care? |
| How is the staffing at the hospital/health centre now? | - Working hours/shifts - Are some days or months busier than others? Why? - Staff availability at night? For emergencies? - Any staff shortages? |
| How often do you have shortages of medicines, rapid diagnostics, or blood products (if available at that facility)? | - Nature of commodity shortage |
| Can you tell me about management of women in pregnancy with malaria, other infections, and nutritional deficiencies  Management? | - Malaria i.e., information about intermittent preventive treatment in pregnancy (IPTp), bed nets - Diagnosis and treatment pathways |
| Can you tell me about management of women in pregnancy with reproductive tract infections? | - Name of infection - Diagnosis and treatment pathways |
| Can you tell me about management of women in pregnancy with anaemia? | - Diagnosis - Management - Other nutrition deficiencies? - Any special needs for adolescent women? |
| Can you tell me about the process that  happens when women come to the facility in labour? | - Where do they present? - How does admission occur? At what stage? - Staff responsible - Paperwork - Examinations performed - Investigations performed - Process for emergencies? - Availability after hours |
| Can you tell me about how women in labour are cared for? | - Who is involved in reviewing the woman - How often is the women reviewed - Frequency of vaginal exams - Who attends the birth - Use of partographs - Are there any guidelines to follow? |
| Who supports the woman in labour?  Do women need comfort in labour?  How do you provide comfort for a  woman in labour? | - How do you manage women having support in labour? Is it possible for a family member to be with her in - labour? - Are there any positions that you think are more comfortable for women to labour in? Are you able to help women get in a comfortable position? - Pain management? - Fluids? |
| Is it possible for a woman to labour with  privacy?  How is privacy provided to a woman in labour? | - Infrastructure, staffing, visitors - What happens if multiple women are in labour? |
| Can you tell me about what happens  immediately after a baby is born? | - What care is provided to the newborn? Specific questions will be asked on: positioning of the baby, drying of the baby, timing of cord clamping, initial checks of baby’s health and pathways when a problem is identified, and process for and timing of initiation of breast feeding - Immediate postpartum care for mother |
| What information do you provide to a woman before and during her labour?  Are there times when additional information is required?  Do you face any challenges in communicating with women? | - Progress of labour, complication, need for episiotomy, assisted delivery, caesarean - Any language barriers? - Do you speak to the woman? Her family? Or both? - Who makes decisions in case of any complications or emergencies? - Consent process - Any common requests or demands from women or their families regarding childbirth or care? |
| In your experience, what do women seem to value most and feel satisfied with in terms of care provided? | - Most important consideration while providing care? - Why do families choose to deliver at this facility? - Is it ok for men to want to be involved in labour? Is it possible at this facility? What are the challenges that are faced if men want to be involved? |
| Can you tell me about the referral pathways between your hospital and other hospitals?   - For mothers - For newborns | - For referring a case - Escalation and referral processes when specialist care is needed - Receiving a referral - Government and catholic - What types of cases are transferred - Are there any forms required to transfer / refer? |
| What does quality maternal care mean to you? |  |
| What does quality newborn care mean to you? |  |
| What would make it easier to provide quality maternal and newborn care? | - Potential enablers in context, health teams and management, enabling environment and support services - Potential of different service planning, including integrated services and varying modes of outreach |
| What makes it challenging to provide quality maternal and newborn care? |  |
| What strengths do you see in your health facility that supports providing quality maternal and newborn care? |  |
| Has there been any significant changes to maternal and newborn care in your facility in the past three years? here?  If worked elsewhere within last 3 years: Are there any aspects to care, you have seen elsewhere that you think is good and should be done here? | - What has been the most significant changes or outcomes - Why the participant chose that change |
| Is there anything that used to happen that you think should be brought back?  Is there anything that is being done now that should be stopped? |  |
| Do you have any suggested improvements for quality of routine and emergency maternal and newborn health services? | - Can you give some short-term improvements and what you think they could achieve? - Can you give some long-term improvements and what you think they could achieve? - How do you think the change you suggested could work? |
| Would you be interested in learning more about quality maternal and newborn care?  Where do you normally look for information on how to provide good maternal and newborn care? |  |
| Would you be interested in attending a  workshop on quality maternal and newborn care? |  |
