# Supplementary material for: “If I am alive, I am happy”: Defining quality of care from the perspectives of key maternal and newborn health stakeholders in Papua New Guinea
Source: PLOS Glob Public Health. 2024 May 21;4(5):e0002548. doi: 10.1371/journal.pgph.0002548 (PMC11108204; doi:10.1371/journal.pgph.0002548)
Supplement: Attachment — Submitted filename: 2024_03_29 Response to reviewers FINAL.docx [file pgph.0002548.s005.docx]

***“If I am alive, I am happy”: Defining quality of care from the perspectives of key maternal and newborn health stakeholders in Papua New Guinea –* Response to Reviewers**

| **#** | **Reviewer 1 Feedback** | **Our Response** |
| --- | --- | --- |
| 1 | This article presents findings from a survey of stakeholders in maternal and newborn care in Papua New Guinea. A suitable number of postnatal women, partners, care providers and administrators from a range of facilities were interviewed. It is perhaps not surprising that health system users and providers have somewhat different orientations when it comes to defining “quality care”, and this finding is not very different from similar published work from sub-Saharan Africa, but this does appear to be the first study of its kind from the Western Pacific region. | Thank you for reviewing this paper, for your kind words, as well as the constructive feedback below. It has helped us refine and improve the paper.  We agree that the findings are in line with previous research in other comparable low-resource regions. However, it is important that this research took place to understand the specific needs of stakeholders in PNG and more broadly, the Western Pacific. |
| 2 | In many places it is hard to dissect out what are the desired standards of care which are not being met, from more general statements about what a facility should offer. While some quotes and researcher comments make it clear in places in others it is not. Of the participants, how many mentioned what would be considered gaps in quality of care? | Thank you for drawing this to our attention. Every participant mentioned some form of gap in the quality of care during their interview. For some participants, especially women and their partners, sometimes this was in the form of a recommendation as to what the health service should be providing, usually because they did not receive that aspect of care in their maternity care experience.  We have tried to clarify this where possible. However, it should be noted that, the goal of our project was to find what stakeholders define as quality of care - not whether or not the facilities were meeting the needs of women/partners/professionals or not. So it’s more about what the desired standards of care are rather than whether or not they are being met. |
| 3 | Related to this the last line of the conclusion should be modified: “Participants believe that the current standard of care does not align with how they define quality of care”. There are examples throughout of individual participants highlighting shortcomings but no clear summary question that this refers to. | Thank you for this comment. Every participant highlighted shortcomings in the quality of the care administered or received when discussing their definitions of quality of care. Therefore we have changed this to read “There is a gap in how stakeholders define quality maternal and newborn healthcare, and the quality of the care which is administered and received.” This is in relation to our aim of defining how stakeholders define quality of care in East New Britain. |
| 4 | The authors bring in new topics in the discussion such as lack of cleaners and work burdens. These should be mentioned in the results. | Thank you for highlighting this. We have now outlined that hygiene and respectful care were compromised due to poor staffing levels, and that staff experienced significant work burdens, as part of *Theme III – What the service possesses* (page 15). |
| 5 | Some of the statements in section 4 seem to rather work against all the previous sections in determining what is perceived as quality of care. This even refers to the “grab” in the title, which seems not to be the greatest choice to support the authors’ contention. Perhaps the authors could do more to address the nuances around this area- i.e. if the pregnancy outcome is good that is all that matters vs the numerous shortcomings outlined in the first three sections. | Thank you for this comment. We have added a few sentences in the discussion navigating this potential conflict. Some participants were primarily concerned with the outcome of a healthy baby/mother, which is understandable given the high mortality/morbidity in PNG. However, this does not take away from the other elements defined as quality care in themes I – III, as these are important for holistic, comprehensive, and high quality care. Theme IV does however demonstrate the severity of the situation in PNG’s maternity sector, and the profound impact it has on all stakeholders. |
| 6 | The authors acknowledge the lack of women who did not attend antenatal care in their sample, but this does limit the utility of their study. | We only included women who attended the facility for birth (rather than ANC) and we have acknowledged this as a limitation. Despite this, we feel the study does have utility and provides much needed information that is highly relevant in PNG. |
| 7 | There are points in the results where the authors use the word “patient” inappropriately including to describe women who never attend antenatal care. Better to find another term whether it be “pregnant women” or something else. | We appreciate this piece of feedback. All instances where the word “patient” was used have been changed to “woman/women”. |
| 8 | Page 5 Line 3: which levels of facilities does this refer to, presumably not Health Posts? | This statement refers to all facilities at all levels, as per the guideline referenced. However, this can be difficult for smaller health services, especially health posts. This has been clarified in the text. |
| 9 | Supporting information in the shape of interview questions are provided. Some are highlighted in yellow, others not. What is the difference? Were these data collected as part of a multi-component survey of which quality of care is just one part? | Thank you for bringing this to our attention. The highlighted portions of text were the questions which were planned to be focussed on in the qualitative analysis. However, during the analysis process, it became clear that there were portions of the transcript which were relevant to the study’s objectives outside these questions. The yellow highlighted text was left as an oversight, and has now been removed.  This study is a subsection of the overarching ‘Healthy Mothers, Healthy Babies” project. This has been clarified in the text on page 4. |
| 10 | The timing of the study included the COVID pandemic. Were any allowances made for this in either study design or implementation, or in data interpretation? Staffing issues specific to the pandemic may have been operating. | Thank you for this comment, you are absolutely correct that Covid had a profound effect on health systems worldwide, including PNG. The data used in this project were actually collected from Sept 2019 and Feb 2020. The Dec date was an error, as other components of the overarching Healthy Mothers Healthy Babies project ran until then, and the lead author made an error with the date. This has been corrected. |
| **#** | **Reviewer 2 Feedback** | **Our Response** |
| 11 | Thank you for allowing me to review this manuscript. It is an important topic with real-world implications and actionable recommendations. It is overall well written. | Thank you for reviewing our paper, for your kind words, as well as constructive feedback that helped us strengthen this paper further. |
| 12 | Pg. 4, paragraph 2, third to last sentence on page – I believe “10,0000” is meant to be “10,000” please double check if these measures are 10,000 or 100,000, but either way, clarify this number. | Thank you for this pick-up. 10,000 is correct, this has been changed. |
| 13 | Pg. 6, last paragraph – interviews were conducted between September 1, 2019 and December 31, 2020. This, of course, was at the start of/during the COVID-19 pandemic. It makes me wonder what the COVID-19 context was in PNG at this time. What were the case counts? Were there policy restrictions? Were practices changed during this time? And, would any of these changes affect care or the answers provided for this study? It would be good to set the context to understand these since quality of care may have been different during the pandemic than at other times. For example, maybe there were restrictions affecting companionship, maybe women did not like wearing masks during their time in the hospital, maybe healthcare professionals were more overwhelmed than usual, or maybe cleaning staff were not going to work for fear of COVID-19 and affecting hygiene – I don’t know any of this since nothing is described about the effects or status quo during COVID-19. It does seem odd that COVID-19 is not mentioned at all in the paper by the authors or by the participants since care in general changed significantly during this time in many parts of the world. | Thank you for this insightful comment. You are absolutely correct that there would have been significant changes in the health/policy landscape around this time. This date is an error. The data were actually collected from Sept 2019 to Feb 2020, with other components of the overarching Healthy Mothers Healthy Babies Study running through to Dec 2020 (which is where this date has come from). This is why there was no mention of the pandemic in any interviews/throughout the paper. We apologise for this error – this has been clarified in the text. Additionally, we have added a sentence acknowledging the limitation of this paper not covering the impact of COVID-19 on the maternity healthcare system in PNG. |
| 14 | Page 9 – Table 2 – recommend to list the health services in the same order as in Table 1 | Thank you for this suggestion, this has been altered. |
| 15 | Page 9 – Table 3 – What is completed Year 12? Is this with regards to education? If so, I would add education. Also, if education, it surprises me that more women had a 12 year education than the partners (0%). Is this correct? I would find it surprising that women would be more educated than men in PNG. If so, does this reflect women who would likely deliver at a facility? Please clarify. | This is a very insightful comment. “Completed Year 12” does refer to education. This has been clarified in the table. We agree that 46% is high considering the broader female population have a year 12 completion rate of 7.8%, and males 9.8% in PNG. This demonstrates that women with this level of education seem to be more likely to attend a facility birth. This has been acknowledged in the limitations of the study. |
| 16 | Page 10 Table 4 – I do not see “antenatal and intrapartum services” in the figure | ‘Antenatal and intrapartum services’ are part of Theme I: Ensuring Access, listed in the right hand column (Sub-themes) in Table 4, Page 10. It has also been added to Figure 1. |
| 17 | Page 11, paragraph 2, sentence 3 quote – recommend to remove “-Partner” at end since already stated and it is the only quote with this descriptor. Recommend to either remove here or add descriptors to all quotes with sites. | Thank you for this note, this has been removed (as well as other occurrences in the text). |
| 18 | Page 14 – mid-first paragraph – it sounds here that participants, including healthcare managers and professionals, are stating that sanitary items are important. Yet, in the figure, “hygiene” is only listed under women. Further, in the discussion, on page 17 (last paragraph, second to last sentence), you discuss hygiene from the perspective of professionals or “staff” as caring about hygiene as it increased their workload. I would re-consider whether “hygiene” is only cared about or mentioned from women as your evidence points to other stakeholders caring about hygiene. | Thank you for this comment. Upon reflection, this is evidence enough that healthcare managers and healthcare professionals include hygiene as an important aspect of quality of care. This has been amended in Figure 1. |
| 19 | Pg. 15, second paragraph, first sentence – I don’t see training in your subthemes or in the figure, yet, it seems to be important. It also surprised me that “guidelines” only fell under administrators and managers, as it would seem professionals would want to have guidelines and training to provide good care. Maybe guidelines here are describing facility policies only (I don’t think so)? If clinical practice guidelines, I would likely change “guidelines” to “Guidelines and training” and move it to the overlapped circle that includes administrators, managers and professionals, as long as professionals talked about standard of practice, which includes guidelines and training. These may also just fall under “best practice,” in which case, I would recommend to move “guidelines and training” to under “best practice” and explain these as part of best practice. Depends on what your data show how to organize this | This is a very insightful comment. Guidelines refers to clinical practice guidelines in this instance. In the raw data, healthcare professionals discussed complication management pathway guidelines, which was missed in the original thematic analysis. We discussed this with members of our team who work/have worked in maternity care in PNG, and they certainly have highlighted the importance of guidelines to their practice. Your suggestion to merge guidelines and training is a good one, and this has been altered in both Table 4 and Figure 1, and now comes under administrators, managers, and professionals. |
| 20 | Pg. 17, second paragraph, sentence 3 – This sentence states that professionals were concerned about timely care, yet “timely care” is shown in the figure as only important to women and their partners. I would recommend to move “timely care” to the part of the diagram where women, partners and professionals overlap. This is also not discussed in the results section, but it is brought up in the discussion. Make sure not to introduce new concepts in the discussion section that have not been brought up in the results section. (e.g., hygiene and timely care). Double check that all concepts brought up by different stakeholders are reflected in the analysis and figure. | Thank you for this comment. This is an oversight on our part, as the results section does not clarify which stakeholders discussed this as part of quality of care. This has been amended in the results section, as well as the figure. |
| 21 | Pg. 19, last paragraph – from your analysis and description of the healthcare system, it would seem reasonable to add that short-term improvements may be made by addressing respectful care. Also, would it be feasible to recommend to hire non-medical personnel to help with hygiene concerns? It seems this is quite a concern to women and professionals and the description sounds dire and it would not require much training. | Thank you for this comment. “Addressing” respectful care is tricky, as factors such as strain on the healthcare system and culture play into this, and as such it is difficult to make generalised recommendations – we do not want to get bogged down in specific staff members beliefs/attitudes/actions. We acknowledge your point recommending the hiring of dedicated cleaners, and have included a sentence discussing this in the ‘implications’ section. |
| 22 | Figure 1 - Some suggestions have been made above regarding presentation of findings. In addition, it seems odd that professionals or partners do not care about “communication” as important to quality care. Please ensure this is in fact not mentioned by these groups, especially since “respectful care” (per all stakeholders) is described as entailing elements of communication.    It was not clear to me why best practice, welcoming environment, respectful care and adequate staffing were in the box, but other elements mentioned by all stakeholder groups were in the overlapping circles but outside the box (survival/safety/water/electricity/satisfaction with care/companionship). Please provide a clear reason for what was in or out of the box or rethink putting all these elements into the box. | - Regarding ‘Communication’: Thank you for this suggestion. In our data points, healthcare workers and partners did not specifically discuss communication. This does mean that they “do not care” about it but did not mention in specifically in these interviews (other components of care may have been more important to them), so cannot include it in the diagram. [Another paper](https://bmchealthservres.biomedcentral.com/articles/10.1186/s12913-023-09723-x), part of the Healthy Mothers Healthy Babies project, delves into specific community perspectives in focus groups, and discusses communication in depth: https://bmchealthservres.biomedcentral.com/articles/10.1186/s12913-023-09723-x - Regarding ‘Privacy’: Thank you for this comment, this is a good point. The diagram has been amended to reflect this. - Regarding the box in the figure: Upon reflection, this is a poorly laid out diagram. We have taken into account your suggestions and feedback, and made a significant change to how the figure is presented. It is now in line with the themes and subthemes, and presented as a table, which we believe facilitates better readability and more clearly communicates our findings. |
| 23 | Since the overall writing is well done, I will provide specific spelling / grammar recommendations:  Pg. 2 (second page 2), paragraph 2, sentence 2 – Add coma between “timely” and “equitable”  Pg. 3, paragraph 2, sentence 4 – change “publishd” to “published”  Pg. 6, paragraph 2, sentence 2 – Recommend to change to “All four were experienced maternal and child health researchers, with qualifications in nursing, medicine, and public health; had extensive training in qualitative interview techniques; and had detailed knowledge of the East New Britain healthcare system, though both personal and professional experience.”  Pg. 6, paragraph 2, sentence 4 – remove comma between “asked” and “and”  Pg. 7, paragraph 2, sentence 1 – add commas between “care” and “where” and between “attended” and “was”  Pg. 7, paragraph 3, last sentence – remove comma between “validated” and “and”  Pg. 13, paragraph 3, sentence 6 – recommend to add “on” inside the brackets between “examination” and “us”  Pg. 18, paragraph 1, sentence 2 – recommend to change “assist” to “assisting”  Pg. 19, paragraph 3, sentence 1 – recommend to remove comma between “healthcare” and “and”  Pg. 20, paragraph 2, last sentence – add period at end of sentence  Figure 1 – Hygiene and affordability are spelled incorrectly – please correct. | Thank you for these suggestions to facilitate readability and clarity. All have been reviewed and accepted. |
